# Mendelian randomization of stroke risk after total hip and knee replacements

**DOI:** 10.3389/fgene.2024.1435124

**Published:** 2024-07-11

**Authors:** Liang Pang, Zhihui Zheng, Pingping Su, Zhouhengte Xu, Yirui Chen, Zhicheng Liao, Pengcheng Jia, Xiuling Zhang, Cunxian Lv

**Affiliations:** ^1^ Wenzhou TCM Hospital of Zhejiang Chinese Medical University, Wenzhou, China; ^2^ The Eighth Clinical Medical College of Guangzhou University of Chinese Medicine, Foshan, China

**Keywords:** total hip replacement, total knee replacement, stroke, Mendelian randomization, causality, triple hypothesis

## Abstract

**Objective:**

Previous epidemiological studies have indicated an increased risk of neurovascular diseases in patients following total hip and knee replacements. However, definitive conclusions regarding the increased risk of stroke post-replacement remain elusive. Therefore, we conducted a two-sample Mendelian randomization study to investigate the causal relationship between total hip and knee replacements and stroke.

**Methods:**

We utilized summary data from publicly available genome-wide association studies (GWAS). Data concerning total hip replacements (THR, N = 319,037) and total knee replacements (TKR, N = 252,041) were sourced from the Genetics of Osteoarthritis (GO) Consortium. Stroke-related data were obtained from the International Stroke Genetics Consortium, encompassing any stroke (AS), any ischemic stroke (AIS), large vessel ischemic stroke (LV-IS), cardioembolic ischemic stroke (CE-IS), and small vessel ischemic stroke (SV-IS). Our primary causal inference method was the inverse variance weighted (IVW) approach, supplemented by weighted median and MR-Egger regression as secondary inference methods. We utilized the MR-PRESSO global test for outlier detection, Cochran’s Q statistic to assess heterogeneity, and assessed the multiplicity and stability of our findings using *p*-values from MR-PRESSO and MR-Egger regressions, and the leave-one-out method, respectively.

**Results:**

We identified significant genetic associations between THR and both AS (IVW *p* = 0.0001, OR = 1.08, 95% CI = 1.04–1.12) and AIS (IVW *p* = 0.0016, OR = 1.07, 95% CI = 1.03–1.12). Significant associations were also observed between TKR and AS (IVW *p* = 0.0002, OR = 1.08, 95% CI = 1.04–1.12), as well as AIS (IVW *p* = 0.0005, OR = 1.15, 95% CI = 1.06–1.24).

**Conclusion:**

Our findings genetically support an increased risk of stroke following total hip and knee replacements. However, further studies are necessary to elucidate the specific mechanisms underlying stroke episodes post-replacement.

## 1 Introduction

Stroke is characterized by neurological deficits resulting from cerebrovascular lesions. Depending on the location and extent of the brain injury, stroke presents with diverse clinical manifestations, including motor, speech, and cognitive dysfunctions, as well as other neurological impairments (Tater and Pandey, 2021; Rost et al., 2022). Strokes are primarily categorized into two types: hemorrhagic strokes and ischemic strokes, with the latter comprising over 80% of all stroke cases (Sacco et al., 2013). Ischemic stroke, the most prevalent form, can be further subdivided into large-vessel ischemic stroke (LV-IS), cardioembolic ischemic stroke (CE-IS), and small-vessel ischemic stroke (SV-IS), according to the TOAST (Trial of ORG 10172 in Acute Stroke Treatment) classification (Adams et al., 1993). Recent statistics from the World Stroke Organization (WSO) 2022 report indicate that stroke remains the second leading cause of death globally and the third leading cause of death and disability, measured in Disability-Adjusted Life Years (DALYs). The estimated global economic cost of stroke exceeds $721 billion, representing 0.66% of the global GDP (6). A study projecting the burden of stroke in Europe anticipates a 27% increase in the number of stroke patients within the European Union between 2017 and 2047, further exacerbating the global economic burden of stroke (Wafa et al., 2020). Epidemiological studies have identified stroke as a multifactorial disease influenced by various factors, including age, gender, genetic predisposition, and lifestyle choices. Chronic conditions such as hypertension, diabetes mellitus, and hyperlipidemia, along with unhealthy behaviors like smoking and excessive alcohol consumption, are recognized as significant contributing factors (GBD, 2019 Stroke Collaborators, 2021; Roerecke, 2021).

Total hip and knee replacements are common orthopedic procedures used to treat end-stage hip and knee injuries caused by osteoarthritis, rheumatoid arthritis, traumatic arthritis, and other degenerative joint diseases (Learmonth et al., 2007; Confalonieri and Biazzo, 2019). Surgery becomes the best option when non-surgical treatments (e.g., medications, physical therapy, and lifestyle changes) are ineffective in relieving symptoms (Mancuso et al., 1996; Cross et al., 2006; Dreinhöfer et al., 2006). These surgeries, by replacing damaged joint parts and implanting prostheses, can significantly alleviate patients’ pain, restore the range of motion and function of their hip and knee joints, and greatly improve their quality of life (Ethgen et al., 2004). However, it cannot be denied that total hip and knee replacement as a mature orthopedic technique still has many drawbacks, with the possibility of significant bleeding during and after surgery (Sizer et al., 2015), postoperative deep vein thrombosis (DVT) requiring special attention (Santana et al., 2020), and a certain risk of periprosthetic infections in the postoperative period (Vrgoc et al., 2014). Besides these postoperative complications, total hip and knee arthroplasty can also cause postoperative neurovascular damage. Recent clinical studies have shown that patients undergoing total hip and knee replacement have an increased risk of postoperative stroke (Lalmohamed et al., 2012; Petersen et al., 2019; Richardson et al., 2023). Nevertheless, the results of these observational studies may be affected by confounding factors. To address the common confounding issues in observational studies, the Mendelian randomization (MR) method was developed and has been widely used in epidemiology. Mendelian randomization uses genetic variation as an instrumental variable (IV) to estimate potential causal relationships between exposure factors and outcomes (Smith and Ebrahim, 2003; Burgess et al., 2017a). These genetic variations are randomly distributed during meiosis, minimizing confounding factors and reverse causality, making the MR method particularly effective in simulating randomized controlled trials (Emdin et al., 2017). This methodological advantage enables MR to provide more accurate and reliable causal inferences.

Given the adverse effects of stroke on the health and quality of life of patients and the uncertainty of the potential impact of total hip and knee arthroplasty on the risk of cerebrovascular disease, we conducted a two-sample Mendelian randomization study to assess the impact of total hip replacement and total knee replacement on the incidence of stroke. Through in-depth study and discussion of the associations, we aim to provide new insights and approaches to the perioperative management of total hip and knee replacements.

## 2 Materials and methods

### 2.1 Study design

**FIGURE 1:**
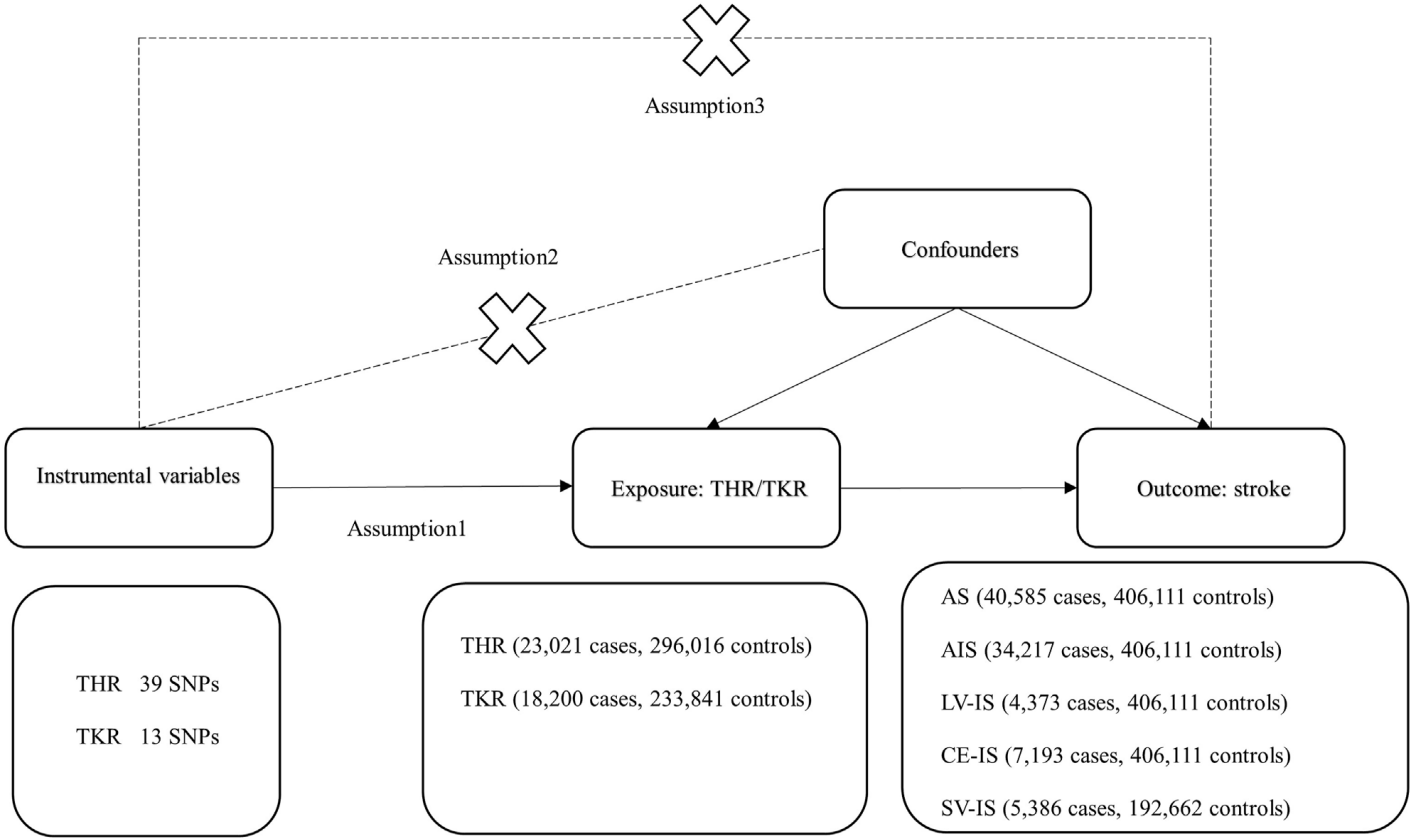
Mendelian randomization basic assumption diagram.

We conducted a two-sample Mendelian randomization analysis to investigate the causal relationship between total hip and knee replacements and stroke incidence. Figure 1 illustrates the basic design of the two-sample MR approach used in our study (Davies et al., 2018). Mendelian randomization, as a statistical method in epidemiology, hinges on three critical assumptions: (a) the correlation assumption, where the genetic variation serving as an instrumental variable must be strongly correlated with the exposure factors; (b) the independence assumption, stipulating that the genetic variance must not be associated with unmeasurable confounders between the exposure and outcome; and (c) the exclusivity assumption, which dictates that the genetic variation affects the outcome solely through the exposure factors, and not directly.

### 2.2 Data sources

Total hip replacement (THR) and total knee replacement (TKR) data were sourced from the Genetics of Osteoarthritis (GO) Consortium, which conducted a genome-wide meta-analysis involving 11 osteoarthritis phenotypes across 13 international cohorts from nine populations. This analysis included summary statistics for up to 826,690 individuals, of whom 177,517 were diagnosed with osteoarthritis (Boer et al., 2021). In our study, THR is defined as total hip replacement for arthritis of the right, left, or both hips, while TKR is defined as total knee replacement for arthritis of the right, left, or both knees.

Stroke data were obtained from summary statistics provided by the International Stroke Genetics Consortium, which published a meta-analysis of genome-wide association (GWA) data for stroke and its subtypes in 2018 (27). The consortium conducted two types of meta-analyses: (a) a fixed-effects meta-analysis restricted to Europeans, involving 40,585 cases and 406,111 controls, and (b) a fixed-effects cross-ethnicity meta-analysis including all samples, with 67,162 cases and 454,450 controls. To minimize bias due to ethnic variations, our study utilized data exclusively from the European-only fixed-effects meta-analysis conducted by the International Stroke Genetics Consortium, encompassing 40,585 cases and 406,111 controls. This data set included any stroke (AS), any ischemic stroke (AIS), large vessel ischemic stroke (LV-IS), cardioembolic ischemic stroke (CE-IS), and small vessel ischemic stroke (SV-IS).

### 2.3 Selection of instrumental variables

In our MR analyses, THR and TKR were treated as exposures, with stroke and its subtypes as the outcomes. For this study, we used single nucleotide polymorphisms (SNPs) that showed genome-wide significant associations with THR and TKR (P < 5E-08) (Purcell et al., 2007) and had no linkage disequilibrium (LD) (*r* ^2^ < 0.001, kb = 10,000) as instrumental variables (Abecasis et al., 2010). Considering that smoking, alcohol consumption, obesity, hypertension, hyperlipidemia, and diabetes are modifiable risk factors for stroke, we searched for SNPs associated with the above risk factors using the LDtrait website and found that three SNPs in THR, rs2510078, rs2820444, and rs75686861, were associated with diabetes, so we excluded these SNPs to mitigate potential confounding effects (Lin et al., 2020). Additionally, SNPs with inconsistent exposure and outcome alleles, as well as palindromic SNPs with moderate allele frequencies, were excluded. We refrained from using surrogate SNPs. After rigorous screening by the above criteria we screened out 39 SNPs associated with THR and 13 SNPs associated with TKR, and only these rigorously screened SNPs were used in the final causal analysis.

### 2.4 MR analysis

In this study, we employed inverse variance weighted (IVW) as the primary method for MR analysis, complemented by MR Egger regression and weighted median (WM) as secondary methods. The IVW method yields the most accurate estimates of causal association effects when all selected SNPs are valid instrumental variables (Burgess et al., 2017b). The WM approach provides valid causal estimates when more than half of the SNPs are valid (Bowden et al., 2016). In cases where all SNPs are considered null instrumental variables, the MR-Egger method offers reliable causality estimates (Bowden et al., 2015).

### 2.5 Sensitivity analysis

Heterogeneity was assessed using IVW and MR Egger regression, with Cochran’s Q statistic employed to quantify its magnitude. Heterogeneity was deemed significant when the *p*-value was below 0.05 (Greco et al., 2015). Horizontal pleiotropy was evaluated by calculating the intercept of the MR Egger regression; the presence of pleiotropy is indicated by a *p*-value of less than 0.05 for the intercept, suggesting that the MR results may not be reliable under these conditions (Bowden et al., 2015). The MR PRESSO global test was utilized to identify outliers, with a *p*-value of less than 0.05 indicating the need to exclude outliers and reanalyze the data in MR (Verbanck et al., 2018). Additionally, leave-one-out analyses were conducted to determine if MR results were influenced by a single SNP. The strength of the genetic instruments was assessed using the F statistic, where an F statistic greater than 10 (F = β^2^/SE^2^) suggests that the MR results are unlikely to be affected by weak instrumental variables (Feng et al., 2022). All MR correlation analyses were performed using R (version 4.2.3) with the TwoSampleMR package (Hemani et al., 2018).

Since multiple tests were conducted, we applied the Bonferroni method to adjust the significance threshold (*p* < 0.05/(2 × 5) = 0.005). To conclude a correlation in this study, the following conditions must be met: (a) a *p*-value of less than 0.005 for the IVW method, (b) consistent results from the weighted median method and MR Egger regression with those of the IVW method, and (c) sensitivity tests indicating that the MR results are not subject to horizontal pleiotropy and are not driven by a single SNP.

## 3 Results

### 3.1 Causal effect of THR on stroke

**FIGURE 2:**
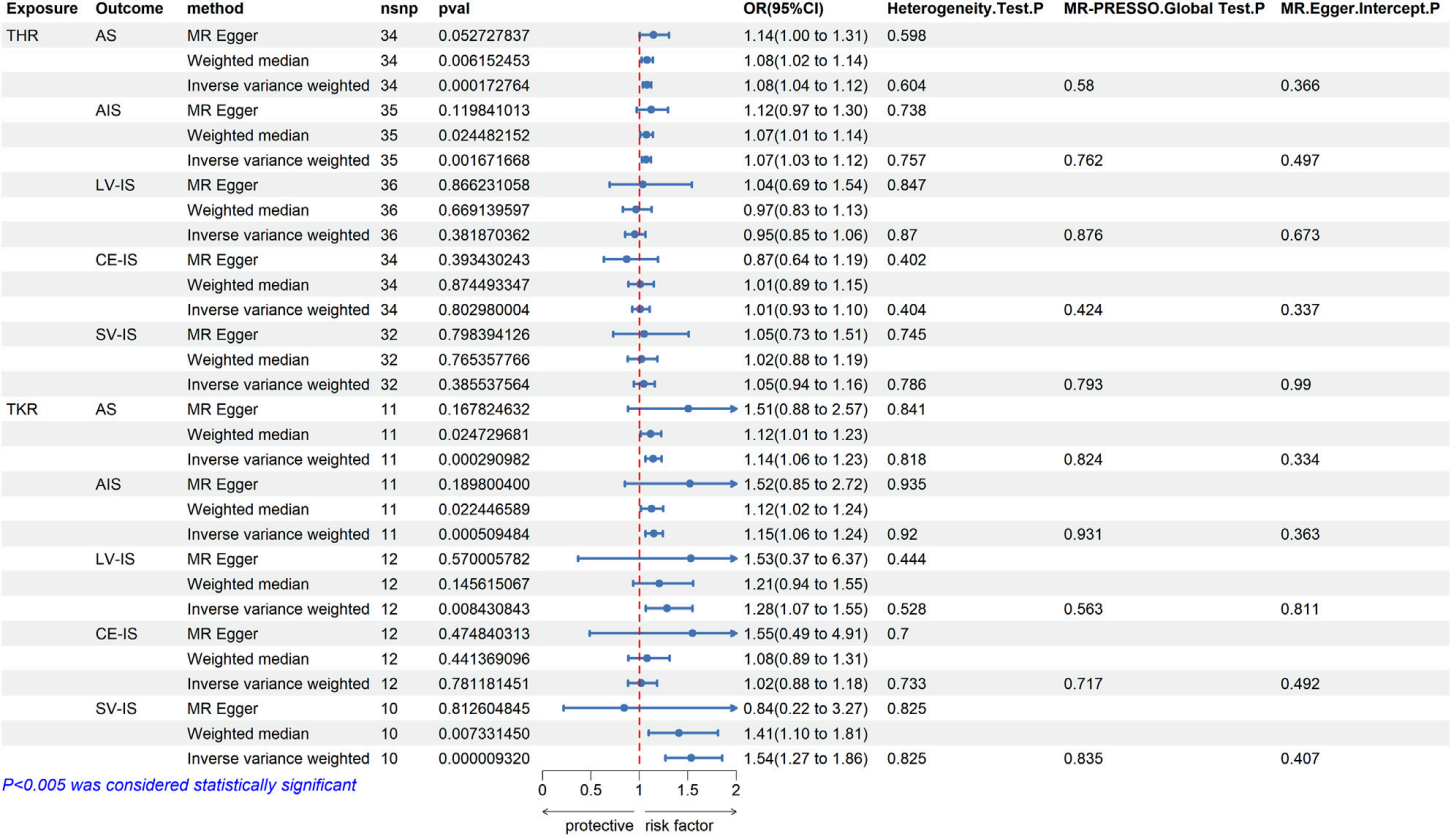
MR estimates from each method of assessing the causal effects of THR/TKR on stroke and its subtypes.

When THR was utilized as exposure data, 39 validated SNPs were identified after controlling for confounding factors such as hypertension, hyperlipidemia, diabetes, smoking, alcohol consumption, and obesity. According to Figure 2, there was a significant genetic association between THR and both any stroke (AS) (IVW *p* = 0.0001, OR = 1.08, 95% CI = 1.04–1.12) and any ischemic stroke (AIS) (IVW *p* = 0.0016, OR = 1.07, 95% CI = 1.03–1.12). However, no significant associations were found between THR and large vessel ischemic stroke (LV-IS) (IVW *p* = 0.3818, OR = 0.95, 95% CI = 0.85–1.06), cardioembolic ischemic stroke (CE-IS) (IVW *p* = 0.8029, OR = 1.01, 95% CI = 0.93–1.10), or small vessel ischemic stroke (SV-IS) (IVW *p* = 0.3855, OR = 1.05, 95% CI = 0.94–1.16). The results from the weighted median and MR Egger methods were consistent with the direction (increased risk) of the IVW method results (see the accompanying material for the relevant scatterplots). The *p*-values for the Cochran’s Q test for both the IVW and MR Egger methods exceeded 0.05, indicating no heterogeneity (see the accompanying material for the associated funnel plots). Similarly, the *p*-values for MR PRESSO and the MR Egger regression intercept were greater than 0.05, suggesting the absence of pleiotropy. Moreover, the results of the leave-one-out analyses demonstrated that the association between THR and stroke was not dependent on any single SNP (see the accompanying material for the relevant leave-one-out plots).

### 3.2 Causal effect of TKR on stroke

After switching the exposure factor to TKR, 13 valid SNPs were identified using the same criteria as for THR. According to Figure 2, there was a significant genetic association between TKR and any stroke (AS) (IVW *p* = 0.0002, OR = 1.08, 95% CI = 1.14–1.23) and any ischemic stroke (AIS) (IVW *p* = 0.0005, OR = 1.15, 95% CI = 1.06–1.24). However, no significant association was observed between TKR and large vessel ischemic stroke (LV-IS) (IVW *p* = 0.0084, OR = 1.28, 95% CI = 1.07–1.55), or cardioembolic ischemic stroke (CE-IS) (IVW *p* = 0.7811, OR = 1.02, 95% CI = 0.88–1.18). Although the IVW results suggested a correlation between TKR and small vessel ischemic stroke (SV-IS) (IVW *p* = 9.32E-06, OR = 1.54, 95% CI = 1.27–1.86), the MR Egger method results did not align with those of the IVW method. The results of the weighted median method and the MR Egger method for the other four-stroke types were consistent with the IVW method, indicating an increased risk (see the accompanying material for the associated scatter plots). The *p*-values for Cochran’s Q test were greater than 0.05 for both the IVW and MR Egger methods, indicating the absence of heterogeneity (see the accompanying material for the associated funnel plots). Additionally, the *p*-values for the MR PRESSO and the MR Egger regression intercept were both above 0.05, suggesting no pleiotropy. Furthermore, the leave-one-out analyses confirmed that the association between TKR and stroke was not dependent on any single SNP (see the accompanying material for the associated leave-one-out plots).

## 4 Discussion

To the best of our knowledge, this is the first study to employ a two-sample MR approach to explore the causal relationship between total hip and knee replacements and stroke. A prior MR study thoroughly examined the causal link between osteoarthritis and stroke, suggesting that hip osteoarthritis may increase the risk of stroke, while no clear causal association has been established between knee osteoarthritis and stroke (Zhao et al., 2022). This observation piqued our interest, as patients with severe hip and knee arthritis frequently undergo total hip and knee replacements. We speculated that these surgical interventions might further elevate the risk of stroke. The findings from our study support this hypothesis, confirming that total hip and knee replacements may indeed be potential risk factors that increase the risk of stroke.

Although our study suggests a potential association between total hip and knee replacements and stroke, it is important to recognize that the specific mechanisms are not yet fully defined. To further explore these mechanisms, we have combined findings from the relevant literature to analyze possible factors for the increased risk of postoperative stroke as well as to provide some new insights and approaches for the perioperative management of total hip and knee replacements. Recent research has identified the use of anticoagulant and analgesic drugs post-surgery as associated with an increased stroke risk. Salášek et al. (2020) discovered that heparin-induced thrombocytopenia could trigger a highly prothrombotic state, potentially leading to arterial thrombosis at various sites, including ischemic stroke. The use of aspirin in combination with celecoxib after total joint replacement may increase the risk of stroke, and the combination of celecoxib + ibuprofen may lead to an increased chance of periprosthetic joint infection (PJI), according to a study by H. Nakata et al. (Nakata et al., 2023). However, according to two studies on the use and safety of aspirin, the use of aspirin for the prevention of DVT after total hip arthroplasty is increasing and its safety profile is not significantly different from that of other anticoagulants (Matharu et al., 2020; Moore et al., 2024). Besides, a study by A. Lalmohamed et al. found that the risk of ischemic stroke was reduced by 70% in patients who used vitamin K antagonists and antiplatelet drugs after surgery, compared to those who did not use any antithrombotic drugs. However, it is worth noting that the study also found that antiplatelet drug users were generally less healthy (Lalmohamed et al., 2012). These findings reveal the complex roles of related anticoagulants and analgesics, and further research could help optimize the selection and administration of anticoagulants and analgesics. Changes in blood pressure post-surgery may also contribute to increased stroke risk; An X et al. found that blood pressure fluctuations in the early postoperative period were associated with stroke and transient ischemia 90 days postoperatively (An et al., 2024). Additionally, certain cardiovascular conditions may act as intermediate factors in stroke occurrence. For example, Sariah Khormaee et al. observed that patients with new-onset atrial fibrillation post-THR or TKR had a 2.7-fold increased risk of ischemic stroke within 1 year, supporting the brain-heart axis theory (Khormaee et al., 2018; Chen et al., 2024). Furthermore, we speculate that the body’s inflammatory response post-surgery might also impact stroke incidence. Total hip and knee replacements, as traumatic surgeries, induce a systemic inflammatory response, activating the immune system, leading to the release of inflammatory factors and infiltration of inflammatory cells (Alam et al., 2018). This systemic inflammatory response may compromise the integrity of the blood-brain barrier, potentially allowing peripheral blood factors to enter the central nervous system and trigger ischemic strokes (Mayer and Fischer, 2024). Understanding this mechanism not only deepens our comprehension of the pathogenesis of postoperative stroke but may also offer new avenues for postoperative risk management.

It is also interesting to note that in addition to the increased risk of stroke associated with total hip and knee replacement, Rafael Robles et al. found in a recent study that the occurrence of stroke also affects the probability of complications after total knee replacement, specifically the risk of PJI, and they found that patients who had a stroke within the 6 months before TKR had an increased risk of PJI at all points in time after TKR, odds are increased, and a stroke within 12–18 months preoperatively increases the risk of PJI 1–2 years after TKR, whereas a period longer than 18 months does not increase the risk of PJI (Robles et al., 2024). Considering the factors mentioned above that may increase the risk of stroke after total hip and knee replacement, we give a few points about the perioperative management of total hip and knee replacement: (a) For patients with a history of stroke, it is recommended that total hip and knee replacement should be performed at least 6 months after the onset of stroke, with an optimal interval of 18 months, to reduce the postoperative PJI risk. (b) Individualized anticoagulation and analgesic regimen according to the patient’s specific situation can reduce the risk of postoperative stroke and PJI. (c) Strengthening postoperative blood pressure management to prevent the risk of stroke caused by blood pressure fluctuations in the early postoperative period. (d) Optimizing postoperative management strategies and reducing the risk of complications through multidisciplinary team care can improve patient prognosis (Rullan-Oliver et al., 2024).

Our study made important findings and has the obvious advantage of having more reliable results and less affected by confounding factors than several previous observational studies (Lalmohamed et al., 2012; Petersen et al., 2019; Richardson et al., 2023). Although all of their results showed that the risk of stroke is increased in patients after total hip and knee replacement, traditional observational studies could not be sure that the increase in the risk of postoperative stroke in patients was only affected by total hip and knee replacement. And cannot completely rule out the influence of certain lifestyle habits and chronic diseases in patients that may contribute to the increased risk of stroke, which is the strength of Mendelian randomization. However, there are limitations to our study, such as the fact that the sample included only European populations; therefore, our findings may not be directly generalizable to other ethnic groups (Howard et al., 2005; Cainzos-Achirica et al., 2019). Additionally, while we have suggested several potential biological mechanisms that could explain the association between total hip and knee replacements and stroke, further laboratory studies and exploration of molecular mechanisms are necessary to elucidate the specific biological pathways involved.

In summary, our two-sample Mendelian randomization study has provided genetic evidence supporting an increased risk of stroke following total hip and knee replacement. Future research could broaden the sample size, include diverse ethnic groups, and delve deeper into the biological mechanisms to fully elucidate the nature of this association.

## Data Availability

The original contributions presented in the study are included in the article/Supplementary Material, further inquiries can be directed to the corresponding author.
