# Supplementary material for: Mendelian randomization of stroke risk after total hip and knee replacements
Source: Front Genet. 2024 Jul 11;15:1435124. doi: 10.3389/fgene.2024.1435124 (PMC11270026; doi:10.3389/fgene.2024.1435124)
Supplement: Supplementary file 1 [file DataSheet1.ZIP › Supplementary_Figures_Result.docx]

**Supplementary Table 1: Sample data sources used in this study and their specific information.**

| Phenotype | Consortium | N controls | N cases | Population studied | Published year | PMID |
| --- | --- | --- | --- | --- | --- | --- |
| THR | GO | 296,016 | 23,021 | European and East Asian | 2021 | 34450027 |
| TKR | GO | 233,841 | 18,200 | European and East Asian | 2021 | 34450027 |
| AS | MEGASTROKE | 406,111 | 40,585 | European | 2018 | 29531354 |
| AIS | MEGASTROKE | 406,111 | 34,217 | European | 2018 | 29531354 |
| LV-IS | MEGASTROKE | 406,111 | 4,373 | European | 2018 | 29531354 |
| CE-IS | MEGASTROKE | 406,111 | 7,193 | European | 2018 | 29531354 |
| SV-IS | MEGASTROKE | 192,662 | 5,386 | European | 2018 | 29531354 |

THR: undergone total hip replacement due to osteoarthritis in left, right, or both hip joints; TKR: undergone total knee replacement due to osteoarthritis in left, right, or both knee joints; AS: any stroke; AIS: any ischemic stroke; LV-IS: large vessel ischemic stroke; CE-IS: cardioembolic ischemic stroke; SV-IS: small vessel ischemic stroke; GO: The Genetics of Osteoarthritis (GO) consortium. These are the genome-wide meta-analysis summary statistics for 11 osteoarthritis phenotypes across 13 international cohorts stemming from 9 populations, comprising up to 826,690 individuals (177,517 osteoarthritis patients). The European population accounts for 78 percent of the total. MEGASTROKE: The MEGASTROKE consortium, a large-scale international collaboration launched by the International Stroke Genetics Consortium, releases the summary statistics from the 2018 meta-analysis of Genome-wide Association (GWA) data in stroke and stroke subtypes to enable other researchers to explore these data for scientific purposes. The MEGASTROKE consortium provides results for the following two meta-analyses as detailed in the original MEGASTROKE publication (Malik et al., Nat Genet, 2018): (1) a fixed-effects meta-analysis restricted to Europeans (40,585 cases; 406,111 controls), and (2) a fixed-effects trans-ethnic meta-analysis including all samples (67,162 cases; 454,450 controls).

**Supplementary Figures**

| 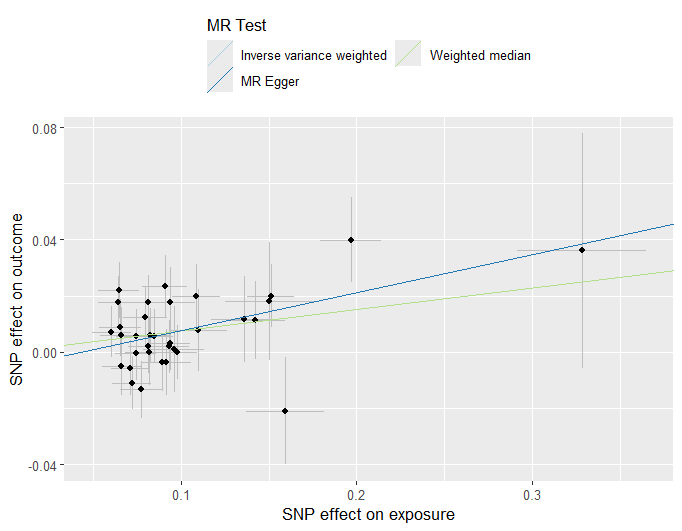  （a） | 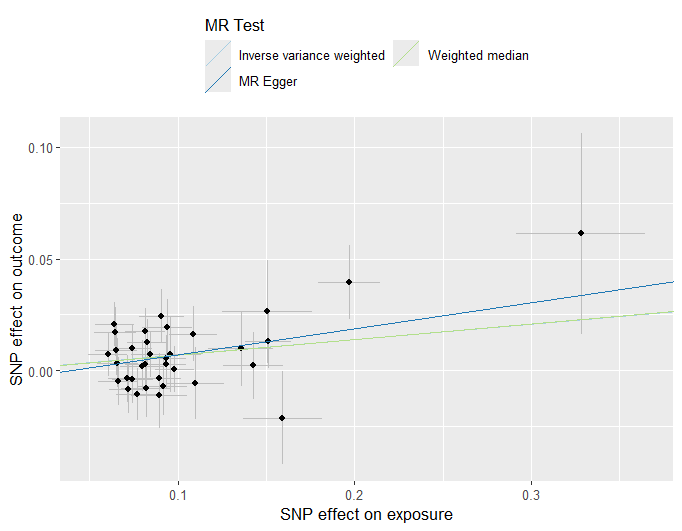  (b) | 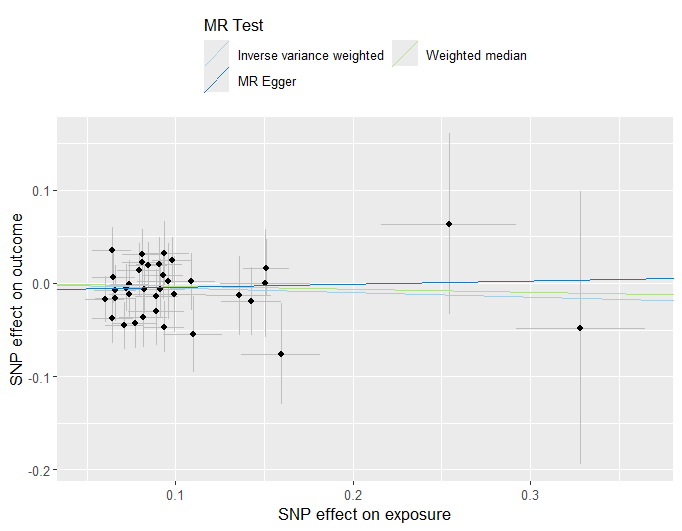  (c) | 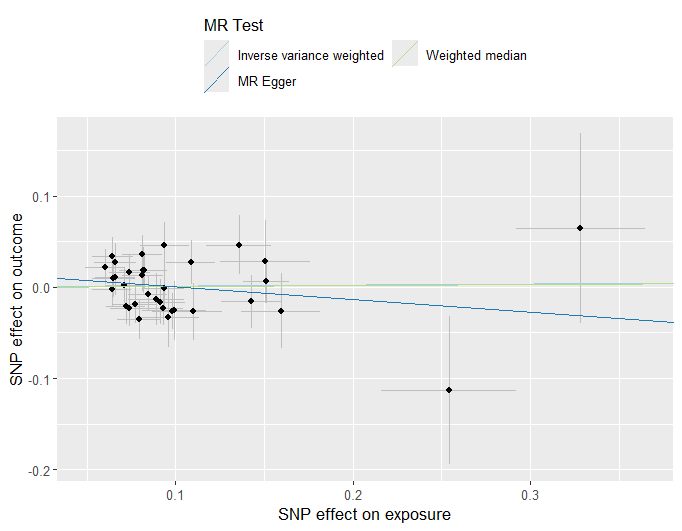  (d) | 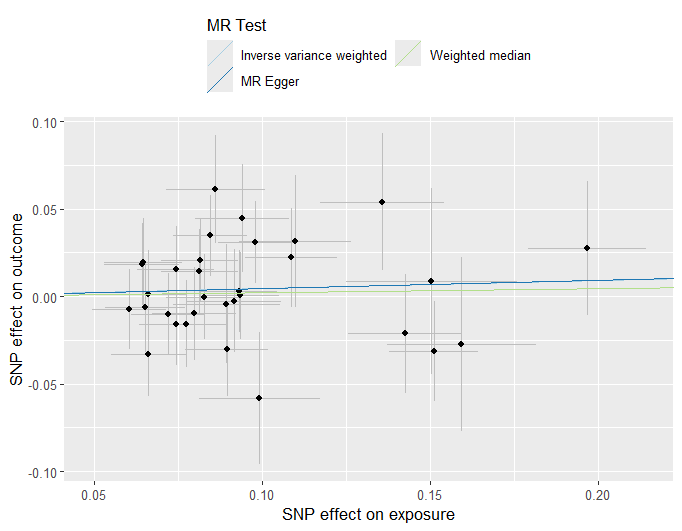  (e) |
| --- | --- | --- | --- | --- |
| 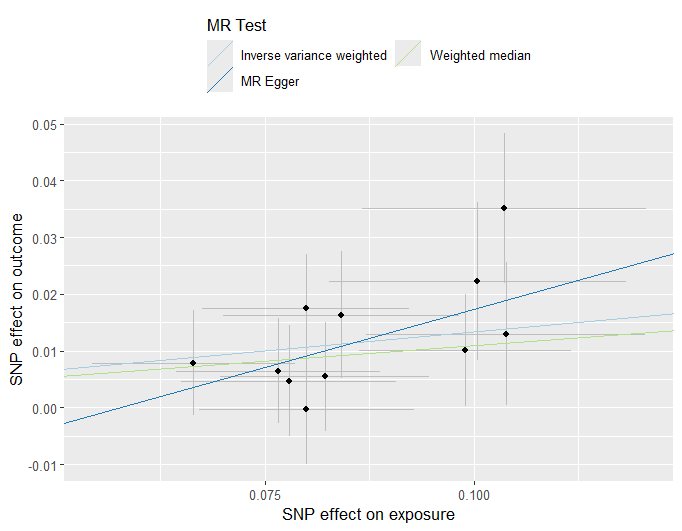  (f) | 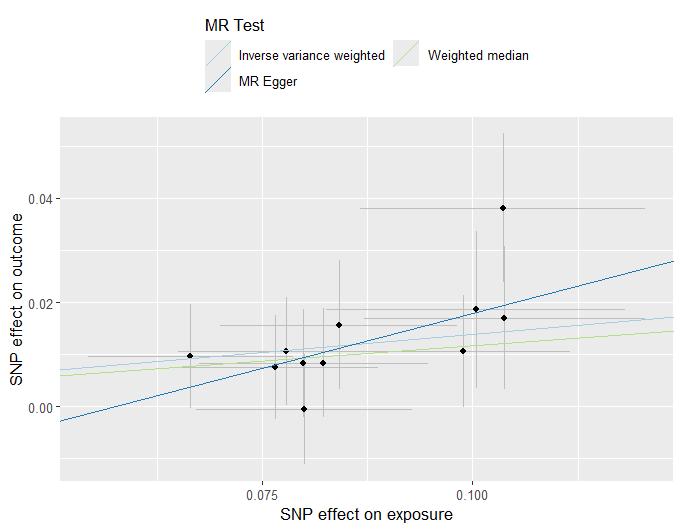  (g) | 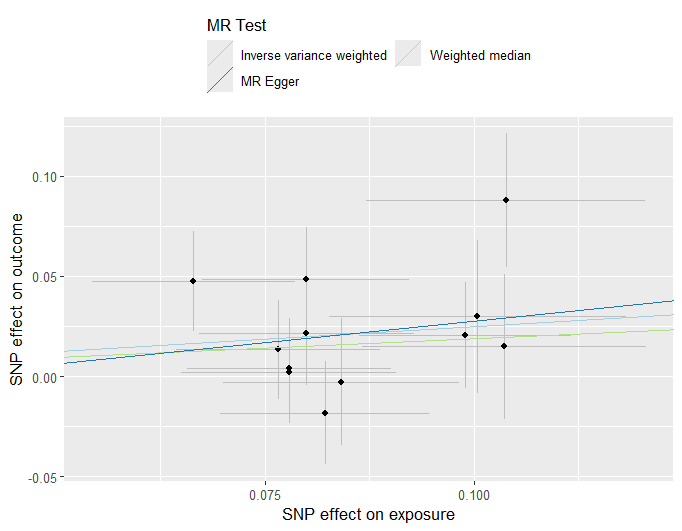  (h) | 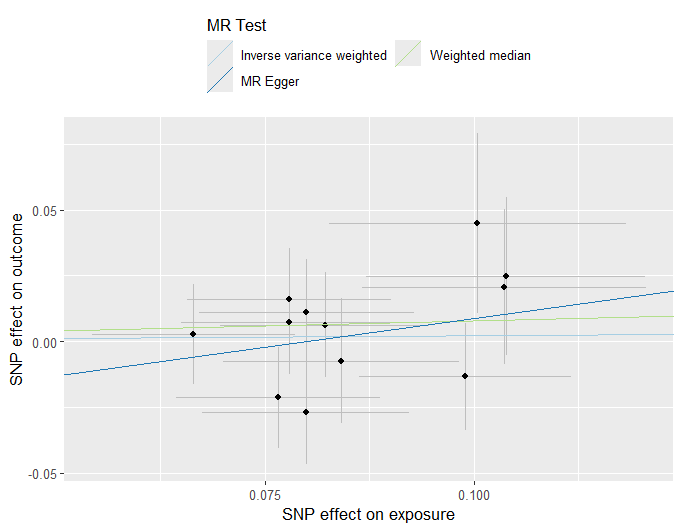  (i) | 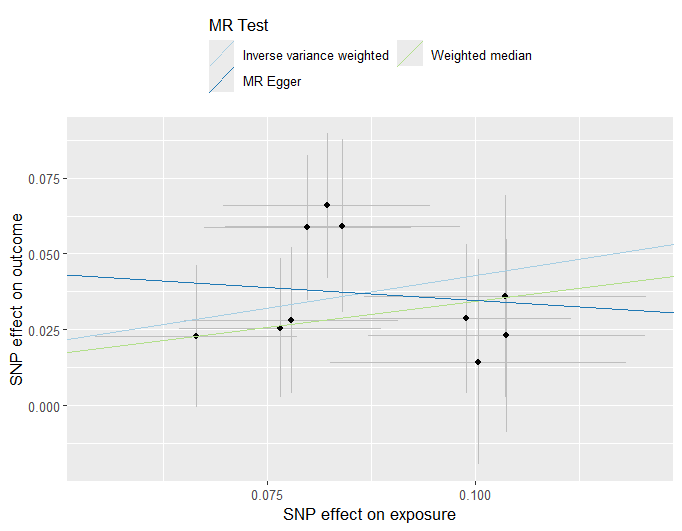  (j) |

**Supplementary Fig.1: Scatter plots illustrating the influence of SNPs on THR and TKR to stroke and its subtypes.** Figures (a-e) represent the relationship between THR and AS, AIS, LV-IS, CE-IS, and SV-IS, respectively. Figures (f-j) depict the relationship between TKR and AS, AIS, LV-IS, CE-IS, and SV-IS, respectively. The x-axis represents the effect size of SNPs on THR and TKR, while the y-axis represents the effect size of SNPs on stroke and its subtypes. The colors of the fitted line correspond to the three approaches used in univariable Mendelian randomization (MR) analyses.

| 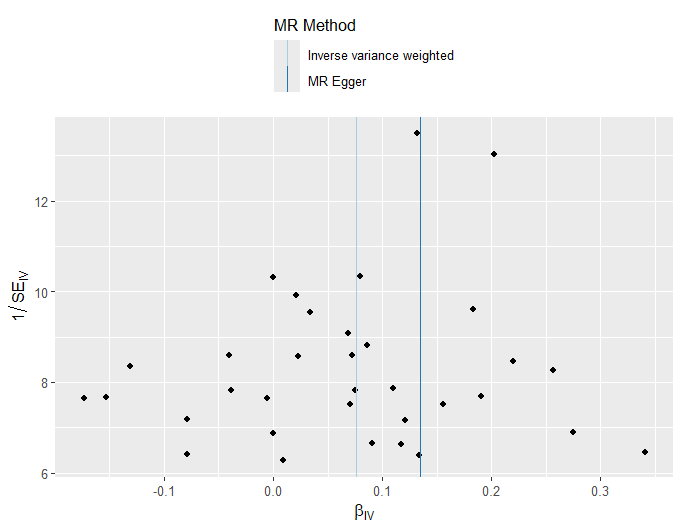  (a) | 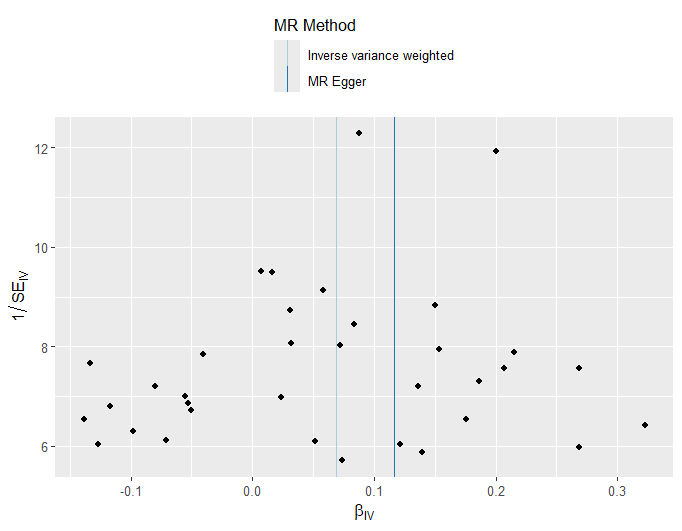  (b) | 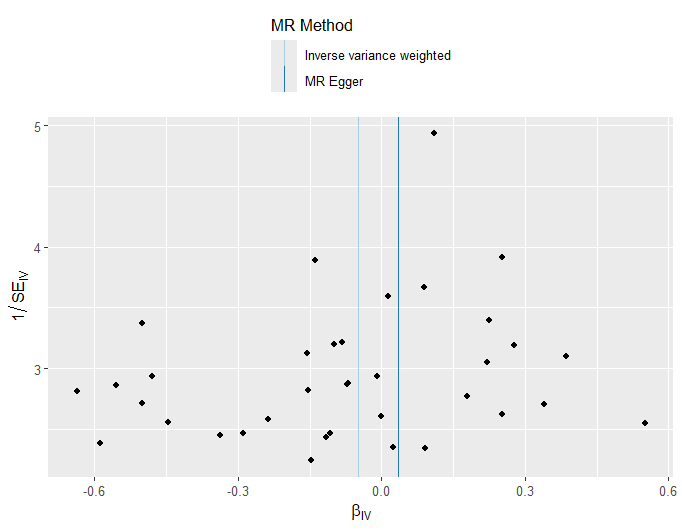  (c) | 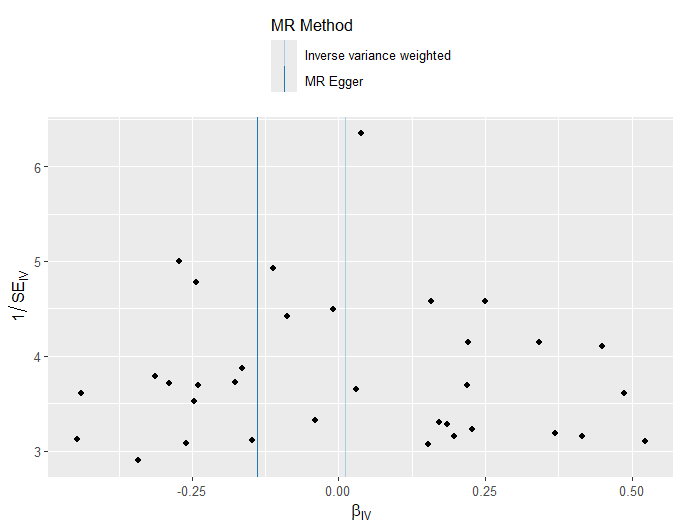  (d) | 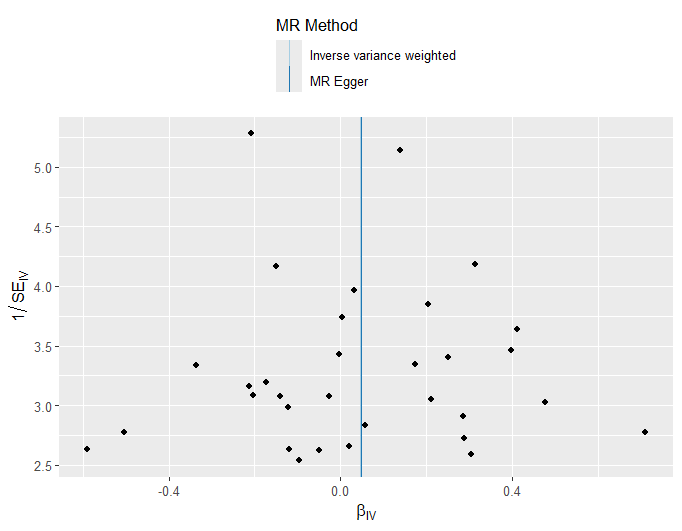  (e) |
| --- | --- | --- | --- | --- |
| 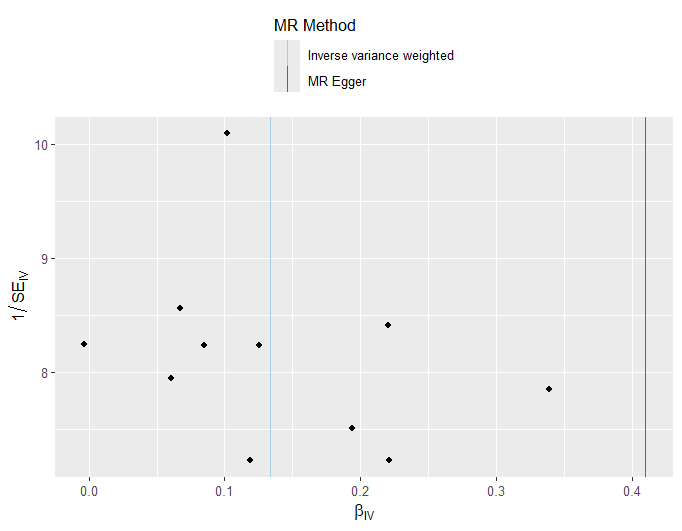  (f) | 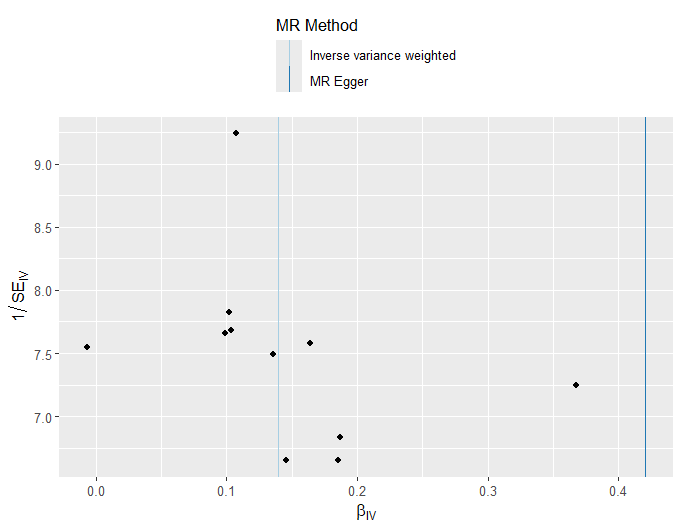  (g) | 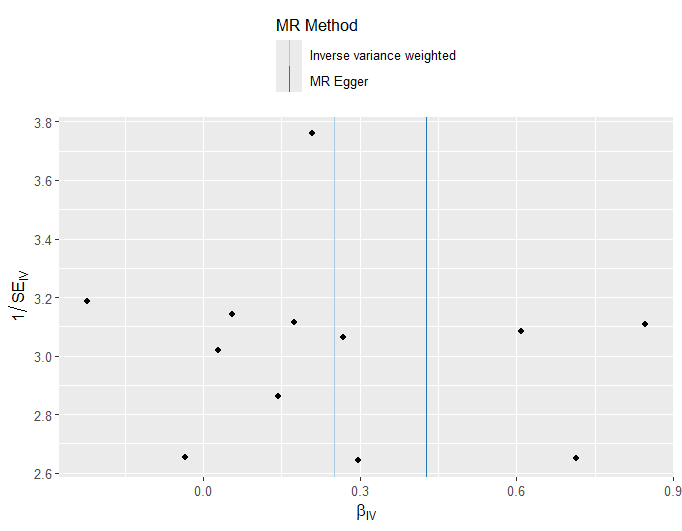  (h) | 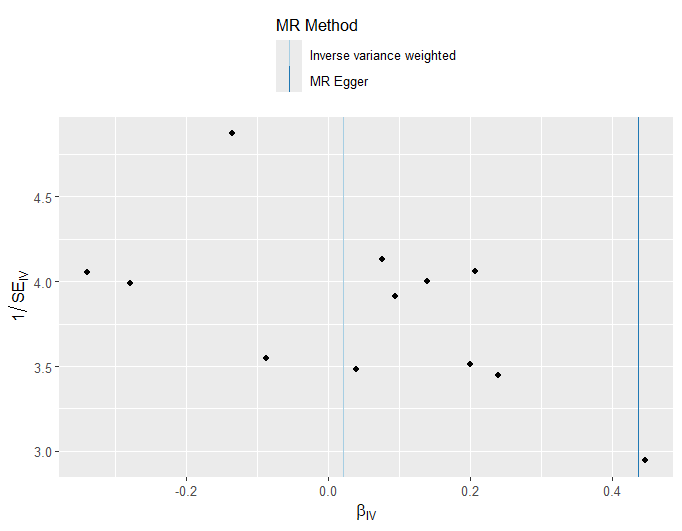  (i) | 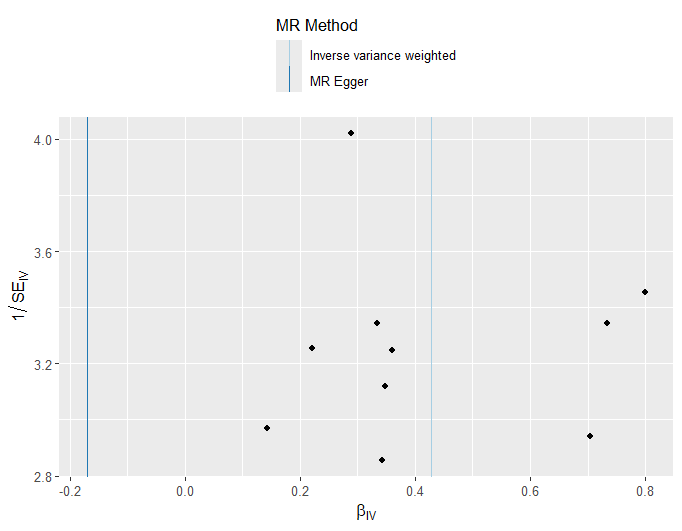  (g) |

**Supplementary Fig.2: Funnel plots illustrating the symmetrical distribution of individual variant estimates around the point estimate.** Figures (a-e) represent the relationship between THR and AS, AIS, LV-IS, CE-IS, and SV-IS, respectively. Figures (f-j) depict the relationship between TKR and AS, AIS, LV-IS, CE-IS, and SV-IS, respectively. The x-axis represents the Mendelian randomization (MR) estimate of individual SNPs, while the y-axis represents the inverse of their standard error.

| 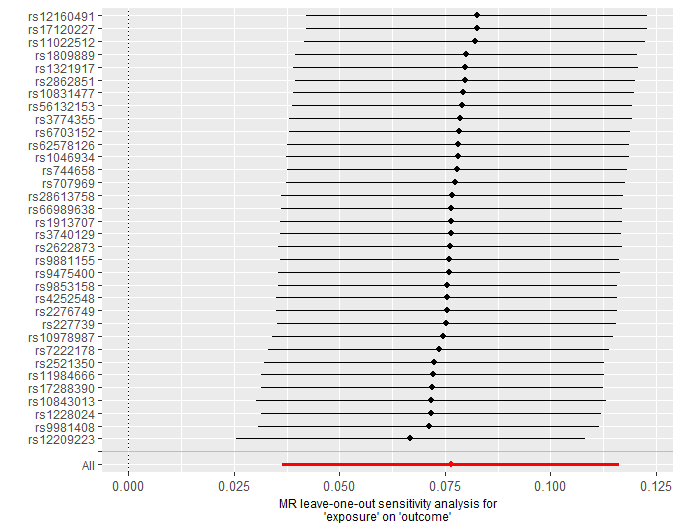  (a) | 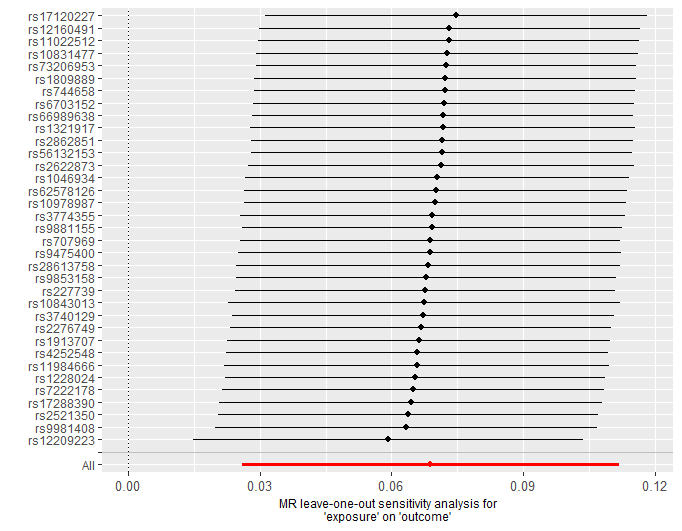  (b) | 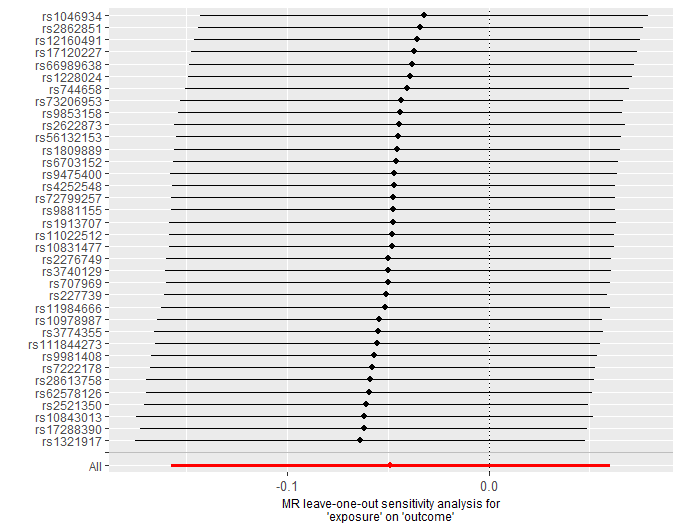  (c) | 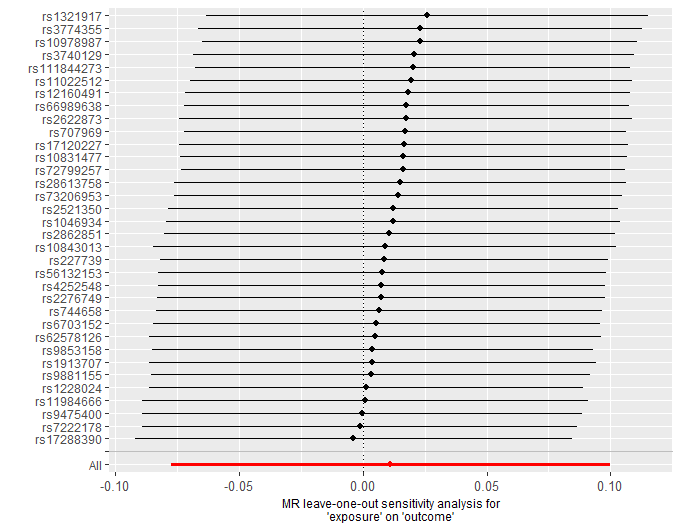  (d) | 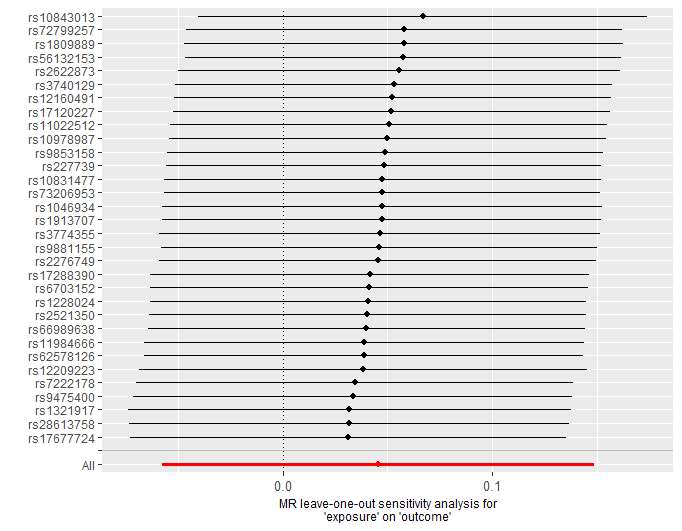  (e) |
| --- | --- | --- | --- | --- |
| 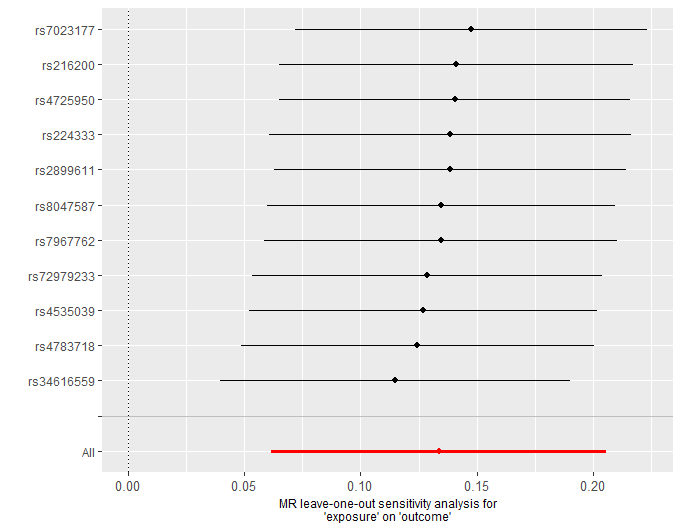  (f) | 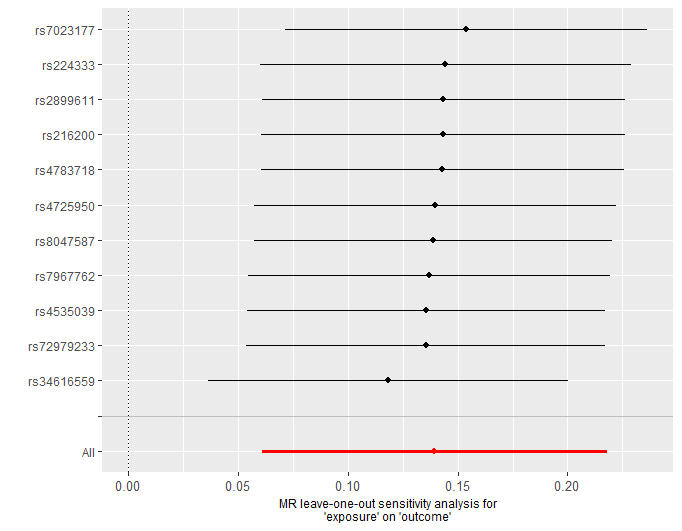  (g) | 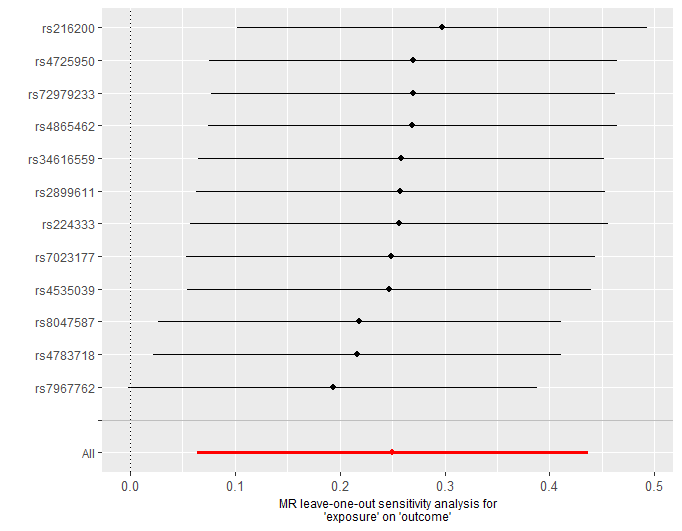  (h) | 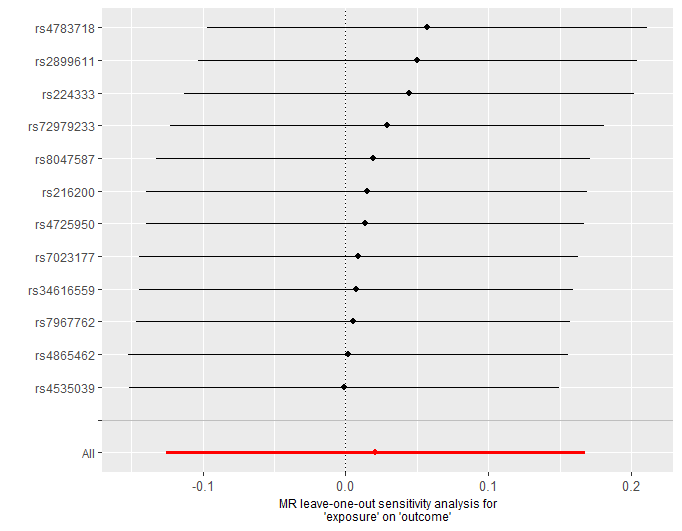  (i) | 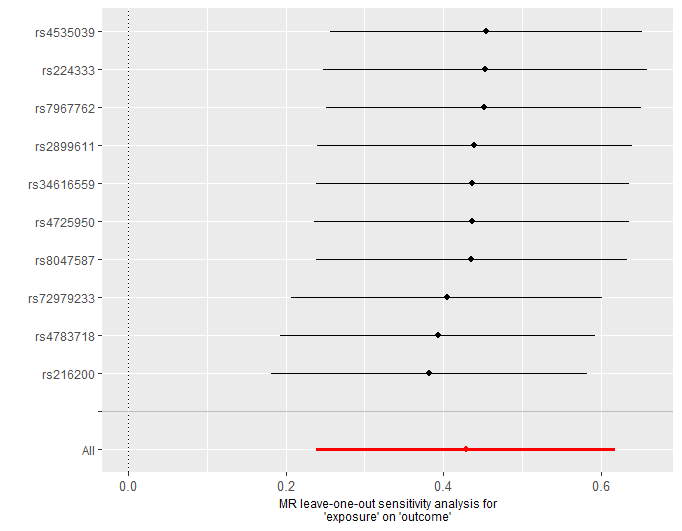  (g) |

**Supplementary Fig.3: The leave-one-out method reflects the stability of the results of Mendel's randomization analysis.** Figures (a-e) represent the relationship between THR and AS, AIS, LV-IS, CE-IS, and SV-IS, respectively. Figures (f-j) depict the relationship between TKR and AS, AIS, LV-IS, CE-IS, and SV-IS, respectively. The red line represents the estimated causal effect calculated using all SNPs, reflecting the average causal relationship between the overall genetic association and exposure and outcome. The black line illustrates the trajectory of estimated causal effects obtained by analyzing the remaining SNPs after each SNP is individually removed. The dotted line is employed to mark the 95% confidence interval boundary of the point estimate, illustrating the range of uncertainty.
